# The VALUE of antibiotic stewardship for companion animals: Understanding appropriate antibiotic prescribing for pet cats and dogs in veterinary clinics in Singapore

**DOI:** 10.1016/j.onehlt.2025.100994

**Published:** 2025-02-11

**Authors:** Huiling Guo, Zoe Jane-Lara Hildon, Lok Hang Wong, Timothy Chua, Boon Han Teo, Angela Chow

**Affiliations:** a Department of Preventive and Population Medicine, Office of Clinical Epidemiology, Analytics, and Knowledge, Tan Tock Seng Hospital, Singapore; b Saw Swee Hock School of Public Health and National University Health System, National University of Singapore, Singapore; c National Centre for Infectious Diseases, Ministry of Health, Singapore; d Singapore Veterinary Association, Singapore; e Lee Kong Chian School of Medicine, Nanyang Technological University, Singapore

**Keywords:** Antimicrobial resistance, Antimicrobial stewardship, Companion animals, Veterinary clinics, Antibiotic prescribing, VALUE model

## Abstract

**Background:**

Understanding the factors influencing antibiotic prescribing for pets can inform future interventions to prevent development and spillover of antimicrobial resistance (AMR) from pets to other animals, humans and the wider environment.

**Material and methods:**

We conducted interviews with 19 veterinarians (January—July 2022) to explore factors influencing antibiotic prescribing for cats and dogs. Thematic analysis was performed using a VALUE model and themes were segmented by consultation touchpoints.

**Results:**

We observed that veterinary clinics in Singapore heavily prioritised business viability. Existing antibiotic stewardship efforts driven by individual veterinarians were often justified as meeting pet owners' satisfaction instead. National guidelines being loosely followed, but AMR-related values and practices were mostly aligned to those of key decision-making veterinarians. Open discussions on antibiotic prescribing amongst different veterinary professionals and shared decision-making (SDM) with pet owners were common. Audits were welcomed by veterinarians but resource limitations were a major concern.

**Conclusion:**

Recommendations to support veterinarians in prescribing antibiotics appropriately for cats and dogs range from formalising antibiotic stewardship as a clinic value, providing collective training for all veterinary professionals, continuing SDM with pet owners, automating tracking of meaningful indicators for monitoring and evaluation, and setting up a feedback system to inform behaviour change.

## Highlights

• An enabling environment, both nationally and within clinics, needs to be created.
• Formalising antibiotic stewardship efforts as a clinic value is highly recommended.
• Collective training for veterinary staff aligns knowledge and antibiotic practices.
• Shared decision-making with pet owners addresses inappropriate use of antibiotics.
• Tracking meaningful indicators, with automation considered, for audit is essential.

## 1 Introduction

In 2019 alone, 4.95 million global deaths were associated with antimicrobial resistance (AMR) [1]. To slow down AMR progression, collective efforts from human, animal and environment health sectors – the One Health approach – are required [2].

Cats and dogs can act as reservoirs to similar pool of resistant bacteria commonly found in humans [3]. They can harbour and transmit antimicrobial-resistant bacteria and genes to their owners through close contact and shared environment [4]. However, there is a paucity of genomic studies that report the transmission dynamics of these bacteria and their resistant genes that provide sufficient evidence to attribute AMR-related infections or colonisation in humans to pet sources. Any probable transmission of resistant bacteria or genes from pets to owners can ultimately reduce the effectiveness of medically important antibiotics in humans [5], further increasing the burden of AMR in human health.

At veterinary clinics, the decision to prescribe antibiotics for pet cats and dogs by veterinarians not only stems on clinical presentations and laboratory results [6], but also complex non-clinical factors, such as business-related concerns [[6], [7], [8], [9], [10]] and veterinarians' attitude towards antibiotic prescribing [11]. Though much is already understood about veterinarians' antibiotic prescribing behaviours, further in-depth and conceptually-informed understanding of decision-making pathways within the diverse veterinary settings, together with better accounting for context [12], are needed. With that in mind, this study aimed to better understand where and how to implement interventions to improve antibiotic prescribing practices for pet cats and dogs in Singapore.

To conceptualise this study, we leveraged on an existing **VALUE** model [13] (see below for expansion of acronym), and a value-driven approach [14] that informs appropriate antibiotic use. The model and its underpinnings were initially conceived based on exploratory studies related to antibiotic prescribing and use in the primary care and community settings, respectively. Here, the VALUE dimensions are extended for use in the veterinary setting for the first time, and they are mapped onto key, known touchpoints along the veterinary consultation journey. Our study objectives are to explore:

(1) Touchpoint #1 (Context): Entry into the veterinary clinic – **V**alues of veterinary clinics and veterinarians with respect to practices related to appropriate antibiotic prescribing.
(2) Touchpoint #2 (Mechanism): Diagnosis and deciding on an appropriate antibiotic treatment plan – **A**lignment between veterinarians on how to diagnose, discuss and move forward to treat according to agreed antibiotic prescribing practices.
(3) Touchpoint #3 (Mechanism): Shared decision-making and antibiotic prescribing – **L**iaison between veterinarians and pet owners.
(4) Touchpoint #4 (Mechanism): Audit and feedback processes – **U**se of monitoring data to enhance evidence-based, performance-driven antibiotic decisions, and **E**valuation to enhance future planning to promote appropriate antibiotic prescribing practices.

Though we consider touchpoints sequentially, we do not assume that decision-making pathways are necessarily linear. Feedback loops and changes in procedures will inform a dynamic overall service delivery system.

## 2 Materials and methods

### 2.1 Study design and sample selection

Semi-structured in-depth interviews were conducted amongst licensed veterinarians practising in veterinary clinics located within Singapore between January and July 2022.

In Singapore, all veterinary clinics are privately funded; the majority being single or small practices. Only two large chains were operating at the time of the study. On average, each veterinary clinic is operated by two to four veterinarians and a mixture of nurses, technicians and administrative staff. All veterinarians, nurses and technicians are trained in either veterinary medicine or veterinary science but only those graduated from selected schools are licensed as veterinarians in Singapore [15]. Every veterinary clinic is managed by a lead senior veterinarian who makes most operational decisions and guides fellow veterinarians through clinical discussions. We defined them as the *key decision-makers* of the clinic.

To achieve maximum variation, we purposively recruited veterinarians who were key and non-key decision-makers from veterinary clinics of single, small and chain practices, with a good mix of gender. Veterinarians licensed as specialists were also separately included in the study. The sampling was anchored by principles of data saturation [16]. This study was approved by the National Healthcare Group Domain Specific Review Board, and informed consent was taken on the day of the interview. Study methods are reported according to the Consolidated Criteria for Reporting Qualitative Research (COREQ) guidelines [17].

### 2.2 Semi-structured interviews

A semi-structured interview guide was designed (Supplementary Material 1), anchoring on the VALUE model [13]. All questions were asked in specific reference towards pet cats and dogs only.

Pilot interviews were conducted with two veterinarians by a female post-doctoral researcher (HG), who was trained in qualitative methods, to test the face validity of the questions within the interview guide. Each interview lasted approximately 60 min; all interviews were conducted virtually by HG or a female research assistant, both trained in qualitative fieldwork. At the start of each interview, the interviewer would introduce herself as a public health researcher with no prior knowledge or training in veterinary medicine or veterinary science to minimise social desirability bias. All interviews were audio-recorded and transcribed verbatim. To ensure anonymity, participants were all assigned with a pseudonym, and any identifiers mentioned during the interviews and captured by the audio recordings were removed from the transcripts.

### 2.3 Data analysis and reporting

Basic descriptive data were tabulated using Microsoft Excel. Qualitative analysis was framed using the VALUE model. A theory-driven conceptual framework [18], building on previous literature, was used to hone the analysis and assess the transferability of the value-driven approach.

During data familiarisation, it was noted that the components of the VALUE model were easily mapped to aspects of the consultation journey [19], and the key touchpoints along this pathway, simply described as (1) entry into the clinic, (2) deciding on the diagnosis and antibiotic treatment plan, (3) shared decision-making and antibiotic prescribing, and (4) using audit and feedback. Mapping the VALUE dimensions to these steps, as per our earlier stated objectives, was seen to enhance the pragmatic application of our findings, helping us to tie these to applied recommendations. Emergent findings were compared between practice type (single/small or chain), function in veterinary practice (specialist or non-specialist), and key decision-makers' accounts against those of non-key decision-makers.

A qualitative analyst (LHW) independently coded all transcripts and coding was undertaken using Atlas.ti 9 (Scientific Software Development GmbH, Berlin, Germany) to manage the data. Six transcripts were coded and formed the basis of a preliminary codebook, which was agreed with lead authors and then applied to the remaining transcripts and expanded accordingly. Consolidated, broader themes are listed out in title form, and supporting subthemes are narrated in *italics*. We have matched these to illustrative quotes housed in Table 2, Table 3, Table 4, Table 5.

## 3 Results

### 3.1 Study participants

A total of 19 in-depth interviews were conducted (Table 1) and the median age was 36 (range 24—58) years old. While there were more female participants (68 %), there was nearly equal representation between the two different practice categories, and decision-making roles. Three specialists were interviewed, out of less than 30 specialists recognised and listed under the Singapore Veterinary Association (SVA). All participants were fully employed, with a small proportion providing locum services.

**Table 1:** Characteristics of participants.

Characteristics of participants	Single or small practice (*N* = 11)	Chain practice (*N* = 8)	Total (*N* = 19)
*Role in clinic, N(%)*			
Key decision-maker	8 (73)	2 (25)	10 (53)
Non-key decision-maker	3 (27)	6 (75)	9 (47)
*Function in veterinary practice, N(%)*			
Specialist	2 (18)	1 (13)	3 (16)
Non-specialist	9 (82)	7 (88)	16 (84)
*Age, years*			
Median (min,max)	35.5 (24,58)	35.5 (26,50)	36 (24,58)
*Gender, N(%)*			
Male	4 (36)	2 (25)	6 (32)
Female	7 (64)	6 (75)	13 (68)
*Ethnic group, N(%)*			
Chinese	10 (91)	6 (75)	16 (84)
Malay	0	0	0
Indian	0	0	0
Others	1 (9)	2 (25)	3 (16)
*Residency status, N(%)*			
Singapore Citizen	10 (91)	6 (75)	16 (84)
Singapore Permanent Resident	0	0	0
Foreigner	1 (9)	2 (25)	3 (16)
*Highest educational level, N(%)*			
Basic veterinary degree	9 (82)	5 (63)	14 (74)
Post-graduate qualification	2 (19)	3 (38)	5 (26)
*Other training background, N(%)*			
No other formal training	4 (36)	4 (50)	8 (42)
Specialisation / Other types of formal training	7 (64)	4 (50)	11 (58)
*Employment status, N(%)*			
Full-time permanent	11 (100)	8 (100)	19 (100)
*Locum status, N(%)*			
Yes	1 (9)	2 (25)	3 (16)
No	10 (91)	6 (75)	16 (84)
*Years in veterinary practice, N(%)*			
≤ 10 years	4 (36)	5 (63)	9 (47)
> 10 years	7 (64)	3 (38)	10 (53)
*Years in current practice, N(%)*			
≤ 10 years	7 (64)	7 (88)	14 (74)
> 10 years	4 (36)	1 (13)	5 (26)

**Table 2:** Themes and subthemes related to Touchpoint #1: Entry into the veterinary clinic, and exposure to shared and personal values on prescribing of antibiotics by veterinarians.

Theme	Subthemes	Illustrative quotes
Veterinary clinics heavily valued prioritising business viability	Antibiotic stewardship was seldom placed as central focus in veterinary clinics	*“I would say that probably antibiotic stewardship was never really discussed.”* - *VP17, chain practice, key decision-maker* *“If you're talking about things like antimicrobial or antibiotic guideline, nothing has trickled down on that.”* - *VP16, chain practice, non-key decision-maker*
	Organisational culture and related values tended towards being client- and service-centric	*“We are a business. We are not a charity. As much as we want to provide the best care for animals, this is our livelihood. We have to pay salaries to people, we have to pay rentals, we have to pay for whole lot of things, so it is a business.”* - *VP04, single/small practice, key decision-maker* *“If you are not making money, you can't feed your staff and your staff needs to eat. And these staff also see monetary reward as a form of satisfaction. So if they are well-remunerated, they are willing to put up with more clients. Rather than if they are not well-remunerated.”* - *VP19, chain practice, non-key decision-maker*
	Efforts to sustain clientele pool included building strong rapport and trust with pet owners, for *e.g.* by enhancing their experience through providing education, and offering a competitive edge	*“Not just services that we provide but we foster a close relationship with the client and it helps us improve in different ways…my clinic has already created quite a name for themselves. So we do have a lot of recommendations from client's friends or family saying that this clinic is good…just based on hearsay and what they googled from the Internet and reading reviews, they will naturally just come to us. So I don't think we find any day where we don't have clients.”* - *VP06, single/small practice, non-key decision-maker* *“One of the mottos we have is to let the client [referring to the pet owner] leave with a smile and also to have learnt something. Whether about the pet, whether about his own family or his own self…They leave with something they've learnt, something to take away with.”* - *VP02, single/small practice, key decision-maker* *“It obviously is a competitive environment and just like in [human] medicine, there are patients who doctor hop…there are also clients who vet hop…how do we get new clients, how do we discourage them from going to another clinic? Our clinic is strong on service quality. We give discounts to people who need discounts. And we have different forms of payments that help people to cope with that.”* - *VP02, single/small practice, key decision-maker*
Personal valuing of AMR and One Health by individual veterinarians was, however, sometimes observed, especially in younger practitioners	Sharing and learning from each other, especially from younger veterinarians, was at times valued	*“I do see the importance of antibiotic resistance for sure, especially when applied to human health. I am definitely onboard…the important role that the veterinary industry plays as well…we are all definitely more conscious of it.”* - *VP03, single/small practice, key decision-maker* *“Every time a younger fresh graduate joins the practice, they bring along the latest practices and information…new ways of doing things and perspectives and the latest guidelines and recommendations…that shared passion and learning things from each other.”* - *VP17, chain practice, key decision-maker* *“It's my personal value because I'm interested in One Health. I'm interested in vet public health and I see the value of it…I pushed very hard for it. I would actually tell the older generation like, ‘Don't use so much of that [antibiotic]. There's no indication for that.’ and they were pretty open about it. As long as I could back it up, as long as I could give them a reason why and what would be better to manage the case.”* - *VP16, chain practice, non-key decision-maker*

**Table 3:** Themes and subthemes related to Touchpoint #2. Diagnosis and deciding on appropriate treatment plans that take account of antibiotic stewardship.

Theme	Subthemes	Illustrative quotes
The importance of chain of command in aligning values on tackling AMR set by key decision makers was notable	Personal values of key decision-makers will ultimately reflect organisational values	*“Because I co-own and I make decisions for the practice, so it stems from my professional beliefs [on how antibiotics should be prescribed]. And then I tried to get that translated down within the organisation. Certainly I stressed that to my staff.”* - *VP04, single/small practice, key decision-maker* *“With the other vets, my personal values, pretty standard values I suppose. Like to have compassion for both our patients and clients, try and do our best for the animal. Yeah, I think it's pretty standard.”* - *VP05, single/small practice, key decision-maker*
	Non-key decision-makers still maintained some autonomy in treatment decisions – though it was clarified that, in theory at least, these should align with organisational values	*“I don't manage how the other vets practise. They are still free to have their own autonomy and choose the way they would like to do it. Of course, within some rules, protocols or guidelines to make sure that we're not overdosing and so forth.”* - *VP18, chain practice, key decision-maker* *“I always tell my team that we just want to do our best for each patient and each client. We can't be perfect but we want to know that we are doing the best to our ability at the particular time. The best that we can do may change from time to time but in terms of upholding standards, I try to make sure that we do our best, we do set a certain level of standard of practice of care.”* - *VP04, single/small practice, key decision-maker*
	Locums had to adapt to other's values	*“They run differently, it really depends on who sits at the top…At the other branch, their standard of practice was slightly different from mine. I just had to adapt to what they were doing…because you can't just show up, take a case and change everything. Sometimes you have to continue what the other vet is doing even though you might disagree.”* - *VP16, chain practice, non-key decision-maker* *“Generally, the whole chain practice is quite - uniform in the philosophy. But there may be a bit of variance here and there. It depends on who set the tone for [each clinic].”* - *VP17, chain practice, key decision-maker*
Emphasis on demonstrating the need for antibiotics was seen as aligning with good antibiotic stewardship practices amid limitations	Taking reference from laboratory-based diagnostics for treatment with antibiotics is the known best practice	*“Diagnostically, you have to prove that there is a need. So as I said, if there is urinary tract infection, you would have to prove to me that there are white blood cells in there. If there is ear infection you do a cytology, you look under the slide to prove that there's intracellular bacteria…Usually there needs to be more than a few clinical signs that support the use of antibiotics. Not just what the client says, what the client feels.”* - *VP10, single/small practice, non-key decision-maker* *“I'm very big on evidence-based practice. So I always do the basic minimum work-up which is cytology. So cytology is taking a sample from the skin and looking under a microscope. If I see that there is evidence for antibiotics orally, I will push for it.”* - *VP16, chain practice, non-key decision-maker*
	But this was not always possible, mainly, because of cost considerations	*“Before you give any antibiotics, do a culture and sensitivity. That'll be the best…I mean that is in the ideal world. We're not in that ideal world. A lot of times, the owners want a quick fix…If the condition doesn't get resolved within two days, doesn't look like there's any significant improvement, you're not doing your job. They will go on to the next vet…if you still don't see a significant improvement, let's do culture and sensitivity.”* - *VP01, single/small practice, key decision-maker* *“I based a lot on my clinical judgement and acumen as well. Like especially if you are presented with something for the first time and it's not a chronic infection. Something presenting for the first time depending on how it would look like – the wound looks fairly healthy and you're able to clean up most of the factors. That you know, then I may not go to a culture. And to be honest, because of cost.”* - *VP03, single/small practice, key decision-maker*
	A lack of locally tailored and taught knowledge for how to assess the need for antibiotics was identified	*“In Singapore, vets that are licensed to practice are either from Australia, New Zealand, UK and US and some areas of Europe. Usually they are already quite well equipped with basic knowledge of antibiotics and their use. For other countries around Asia, South East Asia, then to my knowledge, their training as vets is a lot steered towards large animals still. So, maybe the focus on small animal medicine and [related] use of antibiotics are a little bit less robust.* - *VP04, single/small practice, key decision-maker* *“There's no veterinary teaching hospital here in this country…when you have a teaching hospital, they're usually huge research hubs. If you look at University of California, Davis, and Royal Veterinary College, they published a lot because they're teaching hospitals and they often have training programmes…publishing of information that informs the medical professionals within the field. Then it informs their technical decision-making. I think that's lacking here because you just don't have it.”* - *VP08, chain practice, non-key decision-maker*
	Guidelines on appropriate use of antibiotics in companion animals were described as incomplete and open to interpretation – making alignment with standardised practices challenging	*“We have guidelines for certain things that we have worked over the last decade or so, and treatment principles and regimes which were developed for specific common conditions. The vets are encouraged to question the guidelines and talk to me or my business partner if they don't agree. The guidelines are template for them to base their dispensing decisions on. We encourage them to look for variations in conditions…and not follow it by lock, stock and barrel.”* - *VP02, single/small practice, key decision-maker* *“It's very difficult even within the clinic to make strict guidelines that will prevent or not prevent somebody from doing something…what can and what cannot be used for certain things is very much discretionary…I was involved in writing the guidelines for AVS [Animal & Veterinary Service] but I know the guidelines is not complete and not something that can be solely relied upon.”* - *VP07, single/small practice, key decision-maker*
	Veterinarians spoke of being able to seek advice from their seniors and co-workers on the need, dosing *etc.* for antibiotics	*“The colleagues whom I work with, a lot of them were vets in the Philippines. So they have a lot of experience. I go to them a lot when I need help on things, and a lot of the times they have, not just antibiotics but drugs in general, they will kind of teach me and guide me. But it's my choice to make, whether or not I want to really use that dosage.”* - *VP12, chain practice, non-key decision-maker* *“We usually gather in groups of like four vets, the most six vets…it is more like a learning session during lunchtime…we spend one hour talking, but the more interactive, more in-depth discussion is done one-to-one. We strongly encourage juniors to go to any seniors they're comfortable with, and sit down and talk about a case, or disease, or a drug, how we decide on stuff. Most of the interaction is done during case discussion.”* - *VP10, single/small practice, non-key decision-maker*

**Table 4:** Themes and subthemes related to Touchpoint #3. Shared decision making with pet owners and antibiotic prescribing.

Theme	Subthemes	Illustrative quotes
Poor knowledge and unregulated distribution of antibiotics influenced excessive antibiotic demands and self-administration by pet owners	Poor knowledge was described as the main driver of unnecessary antibiotic demand by pet owners	*“Depending on client's age, client's background, client's attitude, sometimes it can be difficult to tell them why or why not antibiotic is given. From a vet's perspective, it's easy. From the client's perspective, some of them thinks that antibiotic is the cure to everything. Some of them don't use finish the whole course, some of them keep spare ones at home, or even start giving human antibiotics. There's a still a long way for education.”* - *VP10, single/small practice, non-key decision-maker* *“I think a lot of times because social media and the internet is so widely available, they become Dr. Google themselves. They will say things like ‘I read somewhere that this is good, or this is bad’ and things like that.”* - *VP13, chain practice, non-key decision-maker*
	Unregulated sales of antibiotics, by non-veterinary trained groomers, pet shop operators and online shops were noted	*“The more common ones, to my knowledge, will be doxycycline. Doxycycline tends to be used for kennel cough cases or even for pneumonia as well in really young puppies or even kittens. That particular antibiotic, I feel, has been used quite often in the pet shops, and also with owners as well…the pet shop would say that ‘oh, it has been given and administered’. But of course [of what] dosages and duration, they're just not too certain.”* - *VP13, chain practice, non-key decision-maker* *“The puppies and kittens could have an infectious cough for 2 or 3 weeks. They would have come from the pet shop or the breeder, or the person who sold it to the pet owners with antibiotics. We have had cases where owners were told that these are vitamins…This is a problem because they come in with very infectious resistant coughs, or pneumonia and we would have to hospitalise the patient and use the next line of antibiotics. It is hard because…a lot of it has to be based on historical data, what we know of the disease and sometimes there are trial and error.”* - *VP03, single/small practice, key decision-maker*
Yet, it was still not unheard of for pet owners to reject antibiotics	Due to better awareness and better understanding around the topic of AMR	*“I've been practicing for over ten years now in Singapore…a lot of people now are actually keen to avoid antibiotics if not required which is great…I do think generally, the knowledge about antibiotic use is improving quite a lot…most pet owners are now more well informed, they do their research ahead of time.”* - *VP18, chain practice, key decision-maker* *“Pet owners are very well educated these days and they don't want medicine in their animals as much as possible…a lot of them are looking for homeopathy but at least they're willing to try just topicals. And if it really doesn't work, then they'll be like ‘okay, I think it's time for the oral medicine’.”* - *VP06, single/small practice, non-key decision-maker*
Making shared decisions with pet owners on antibiotics was a usual practice	Though, it was also not uncommon that ultimately, veterinarians may be asked to make the final decision for antibiotic prescribing	*“We do have quite a lot of regulars that trust us. So the family will just mostly leave it to our discretion on what to advise, and what to treat the pet with…Some of them cannot decide and they prefer that you just tell them what to do. So, it depends on the person.”* - *VP14, single/small practice, key decision-maker* *“Some of them are indecisive. They don't really know what's best for the animal. So at the end of the day, they will like ‘Oh, I really don't know what to do. What will you suggest?’ Then that's when I will give my firm opinion.”* - *VP06, single/small practice, non-key decision-maker*
	Rapport and trust from pet owners played an important role in this, and in dissuading unnecessary demands for antibiotics	*“Personally, I feel that it has affected it a lot. With the clients I regularly see, there is rapport, there is trust. I don't have to justify to them why I'm making the decisions that I'm making…they don't push if I say ‘no, I don't think it needs antibiotics’. The ones that come in once in a while, or those I do not have a relationship with them or walk-ins, generally they're expecting that quick fix and they're out the door. So, there's always that push for antibiotics.”* - *VP16, chain practice, non-key decision-maker* *“As you practise, more and more, there is that level of trust. So usually, you could say anything, or you don't even have to explain, and they'll just take it, you know. But that kind of relationship is built over years. It's not like, if somebody comes in, it's the first time you see them, then it'll be more challenging to convince them. You have to spend more time to educate them on why we're doing it this way.”* - *VP17, chain practice, key decision-maker*
	A variety of clinical scenarios and treatment options would commonly be presented	*“We believe in the right of the owner to choose the option that best fits in their circumstance…What we are afraid of is that, we make the choice for them and after that if it doesn't turn out well, they will come back and write us a complaint letter.”* - *VP19, chain practice, non-key decision-maker* *“We are very keen on scenario presentations…There are lots of tests each scenario would bring, which means lots of costs each branch of your decision-making would make. The client may or may not want to do this test because of cost or because of past experience. So we paint scenarios to them…the client makes the informed decision so he/she can't turn around and [blame us]…as he/she would have been told the advantages and disadvantages and the potential dangers of doing that.”* - *VP02, single/small practice, key decision-maker*
	Willingness to pay for the diagnostic tests was also commonly discussed	*“Because diagnostics will be more expensive as it's privately funded. Most owners don't want to further investigate because the antibiotics, even if it's private, it's cheaper than you spend hundred over dollars on X-ray. They can just get the antibiotics in maybe less than ten dollars, twenty dollars?”* - *VP11, single/small practice, non-key decision-maker* *“Not in particular with regards to antibiotics. I just think that if they don't have to pay, if it's covered by insurance, they are more willing to spend more to do more diagnostic work-up. But not really specific to drugs or treatment, it's more on the diagnostic work-up.”* - *VP14, single/small practice, key decision-maker*

**Table 5:** Themes and subthemes related to Touchpoint #4. Audit and feedback processes to track and evaluate.

Themes	Subthemes	Illustrative quotes
Both the gains and concerns over audits of antibiotic utilisation were expressed	Seen to provide valuable high-level nationwide analysis to help assess the scale of the problem of inappropriate antibiotic use within the veterinary setting	*“On a national level, it may have some alarming bells. If certain medicines are prescribed excessively when it's not indicated, then that would actually show the actual figures for the whole vet community that we're actually using this much. It's time that we need to reduce…you wouldn't realise what other people are doing until you actually see the numbers for yourself because it might just be normal for your own practice.”* - *VP06, single/small practice, non-key decision-maker*
	Such country level initiatives needed to be thoughtfully implemented – and solutions to problems that may be uncovered be supported by veterinarians on the ground	*“The delivery of this has to be very carefully done, carefully thought through. You cannot come in with guns blazing and start auditing everybody because you want to…you need to have some baseline, a certain type of ‘These things cannot be done.’ We need to establish what these parameters are and then work from that baseline…this must come hand-in-hand with a robust regulatory mechanism.”* - *VP02, single/small practice, key decision-maker*
	Audits were seen as potentially depleting resources further, unless an automated tracking system was created	*“I'm speaking from my point of view, I really don't have a lot of time to do my clinical records, even at work…I would foresee a lack of time and availability to really do this even though I feel that it's definitely an excellent idea in monitoring…if there is like a programme, for example, that can also auto-generate something whenever we prescribe an antibiotic, that would be definitely more helpful instead of us having to manually put in our inputs.”* - *VP13, chain practice, non-key decision-maker* *“It just depends on how intensive the audit is. I'm just concerned about the amount of labour and how time consuming to do it, because we already have a lot of tasks…all the paperwork is already very time consuming.”* - *VP14, single/small practice, key decision-maker*

### 3.2 Qualitative findings

**Fig. 1:**
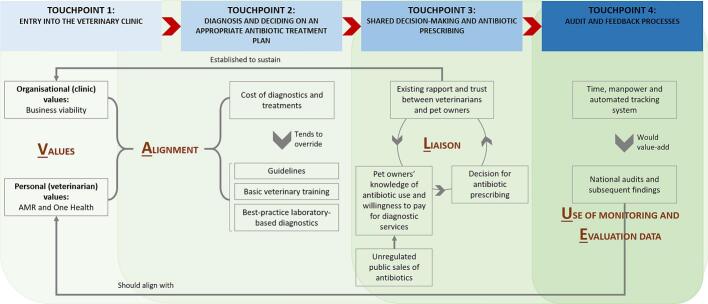
Factors influencing antibiotic prescribing by veterinarians, mapped onto the VALUE model and the key touchpoints identified within the consultation journey at veterinary clinics.

Key findings are summarised in Fig. 1. This schematic provides visualisation of the emerging themes and subthemes, mapped onto the VALUE model and the key touchpoints identified within the consultation journey at veterinary clinics.

<Insert Fig. 1>.

#### 3.2.1 Entry into the veterinary clinic – **V**alues of veterinary clinics and veterinarians with respect to practices related to appropriate antibiotic prescribing (see “touchpoint 1” in Fig. 1)

##### 3.2.1.1 Veterinary clinics heavily valued prioritising business viability

*Antibiotic stewardship was seldom placed as central focus in veterinary clinics.* Purposeful discussions surrounding antibiotic stewardship and guidelines were rare amongst veterinarians within their clinics. Due to the funding model and fragmented nature of the veterinary industry without a public veterinary health service, most veterinary clinics' *organisational culture and related values tended towards being client- and service-centric,* and necessarily focused on revenue generation. *Efforts to sustain clientele pool included building strong rapport and trust with pet owners, for example, by enhancing their experience through providing education, and offering a competitive edge*.

##### 3.2.1.2 Personal valuing of AMR and One Health by individual veterinarians was, however, sometimes observed, especially in younger practitioners

Actively adopting and driving of antibiotic stewardship efforts, and ultimately dialoguing about this in each clinic, were commonly dependent on the individual veterinarian's intrinsic motivation and passion on the topic of AMR, regardless of practice type. This intrinsic trait was particularly notable in younger and/or freshly graduated veterinarians where they strived to bring about changes to improve appropriate antibiotic-related practices in their clinics. *Sharing and learning from each other, especially from these younger veterinarians, was at times valued.*

#### 3.2.2 Diagnosis and deciding on an appropriate antibiotic treatment plan – **A**lignment between veterinarians on how to diagnose, discuss and move forward to treat according to agreed antibiotic prescribing practices (see “touchpoint 2” in Fig. 1)

##### 3.2.2.1 The importance of chain of command in aligning values on tackling AMR set by key decision makers was notable

This was because *personal values of key decision-makers will ultimately reflect organisational values.* This phenomenon was seemingly more pronounced in solo and small practices, possibly due to a lower representation of interviewees who were key decision-makers in chain practices. Nonetheless, regardless of practice type*, non-key decision-makers still maintained some autonomy in treatment decisions – though it was clarified that, in theory at least, these should align with organisational values*.

It was especially evident that *locums had to adapt to others' values* and had the least autonomy and influence in deciding on treatment plans. Locum practices were more common for veterinarians in chain practices, where they cross-covered duties at different locations and to gain wider clinical exposures.

##### 3.2.2.2 Emphasis on demonstrating the need for antibiotics was seen as aligning with good antibiotic stewardship practices amid limitations

*Taking reference from laboratory-based diagnostics for treatment with antibiotics was the known best practice*, as antibiotics should only be prescribed when a bacterial infection is evident. *But this was not always possible, mainly, because of cost considerations*. As a non-subsidised industry with limited insurance provisions, it was emphasised that many pet owners were unable to afford diagnostics and treatments. Furthermore, veterinarians' fear of losing clients was also expressed if these tests, which often were time-consuming and therefore prolonged recovery solutions, had been pushed on them, in contrary to pet owners' desire for a quick fix for their pets. This perception is complementary to the valuing of business viability, as mentioned above, on the need for veterinarians to ensure pet owners' satisfaction in order to maintain a sustainable clientele pool for their clinics. Therefore, veterinarians were limited in what they can do, in terms of conducting cultures and diagnostic tests to determine the presence of a bacterial infection before prescribing antibiotics. Though, it was noted that younger veterinarians were more likely to mention the need to run diagnostics to support selection of antibiotic therapy.

In addition, *a lack of locally tailored and taught knowledge for how to assess the need for antibiotics was identified*. Due to the nature of how veterinarians are licensed in Singapore, they are usually graduates from schools in Australia, New Zealand, the United States and the United Kingdom, where appropriate antibiotic use would be tailored to their individual national guidelines and variety of animal species owned by the local communities. It was suggested by some veterinarians that there was a gap in curricula taught overseas and contextualised antibiotic-related knowledge and practices relevant to Singapore. Problems highlighted included a lack of continuing education provided at the national level for post-graduates, a lack of a veterinary school, and a poor emphasis on veterinary research in Singapore.

In addition, *guidelines (in general; developed at national level or by the clinics themselves) on appropriate use of antibiotics in companion animals were described as incomplete and open to interpretation – making alignment with standardised practices challenging.* With the first local guideline published in November 2022 [20], the veterinarians noted that the guideline was a good start, but could be further improved, for example, supplemented by complex case scenarios to make them more relevant for daily clinical practice.

*Veterinarians spoke of being able to seek advice from their seniors and co-workers on the need, dosing, etc. for antibiotics.* It was shared that veterinary clinics would also have regular meetings between veterinarians to discuss on clinical cases to make joint decisions or for learning purposes. This was an important way to leverage alignment on clinical values as well to standardise clinical practice.

#### 3.2.3 Shared decision-making and antibiotic prescribing – **L**iaison between veterinarians and pet owners (see “touchpoint 3” in Fig. 1)

##### 3.2.3.1 Poor knowledge and unregulated distribution of antibiotics influenced excessive antibiotic demands and self-administration by pet owners

*Poor knowledge was described as the main driver of unnecessary antibiotic demand by pet owners*. This was attributed to some pet owners seeing antibiotics as the cure-all for most infections, having a lack of exposure to proper information, or just lacking education in general. *Unregulated sales of antibiotics, by non-veterinary trained groomers, pet shop operators and online shops were noted*. Pet owners might not be aware of antibiotics used on their pets, therefore making it hard for veterinarians to gauge the animal's prior exposures to antibiotics and prescribe appropriately. Calls were made by some veterinarians for tighter regulations from the local authorities to limit sales from these unauthorised antibiotic distributors.

##### 3.2.3.2 Yet, it was still not unheard of for pet owners to reject antibiotics

*Due to better awareness and better understanding around the topic of AMR*, as observed by some veterinarians, these pet owners often would prefer an alternative solution such as homeopathy, other than antibiotics, to treat their pets.

##### 3.2.3.3 Making shared decisions with pet owners on antibiotics was a usual practice

*Though, it was also not uncommon that ultimately, veterinarians may be asked to make the final decision for antibiotic prescribing*. *Rapport and trust from pet owners played an important role in this, and in dissuading unnecessary demands from pet owners for antibiotics*. For example, it was shared that it was easier to advise and/or to dissuade pet owners against unnecessary antibiotic use when they had a prior clinical encounter with them. Often, veterinarians would *present a variety of clinical scenarios and treatment options*. *Willingness to pay for the diagnostic tests was also commonly discussed* as part of the decision-making process. Shared decision-making (SDM) processes were therefore largely seen to improve satisfaction, rather than as a hindrance.

#### 3.2.4 Audit and feedback processes – **U**se of monitoring data to enhance evidence-based, performance-driven antibiotic decisions, and **E**valuation to enhance future planning to promote appropriate antibiotic prescribing practices (see “touchpoint 4” in Fig. 1)

##### 3.2.4.1 Both the gains and concerns over audits of antibiotic utilisation were expressed

Audits to monitor appropriate antibiotic prescribing were uncommon in veterinary clinics. Interestingly, most veterinarians were in favour of these, as they were *seen to provide potentially valuable high-level nationwide analysis to help assess the scale of the problem of inappropriate antibiotic use within the veterinary setting.* However, it was highlighted that *such country level initiatives needed to be thoughtfully implemented – and solutions to problems that may be uncovered be supported by veterinarians on the ground*. Lastly, it was noted that most veterinary clinics were already heavily stretched with their daily clinical work, and *audits were seen as potentially depleting scant resources (such as time and manpower effort) further, unless an automated tracking system was created.*

## 4 Discussion

### 4.1 Touchpoint #1: Entry into the veterinary clinic

Overall, we observed autonomous, yet unanimous, efforts by veterinarians to ensure appropriate antibiotic prescribing practices for pet cats and dogs. These efforts were very much intrinsically valued by the individual veterinarians, and not especially influenced by the national guidelines [20]. In particular, for veterinarians who could make key decisions within their clinics, they would also actively seek alignment amongst their co-workers to ensure streamlined, appropriate antibiotic prescribing practices were agreed upon and followed. The importance of prudent antibiotic prescribing practices was well acknowledged by veterinarians practising in veterinary clinics in Singapore.

However, an interesting observation was made on how these veterinarians would often attribute good antibiotic-related practices to the purpose of sustaining business revenue for their clinics [11]. For example, efforts to build trusting owner-veterinarian relationships and to provide education to pet owners are known, major strategies to improve appropriate antibiotic prescribing for pets [21] and they were commonly underway in many clinics. Yet, veterinarians had reasoned these efforts as enhancing pet owner experiences to maintain their clientele pool instead, and not leveraged for improving antibiotic prescribing. Embedding specific antibiotic-related messages in these exchanges with pet owners would pay dividends in addressing the AMR issue in the veterinary setting. There is a need to align both individual veterinarians' and clinics' values in AMR by formally recognising and integrating existing antibiotic stewardship efforts into clinic values and missions, that aligns with the clinic's prioritisation of business viability.

Furthermore, the explicit acknowledgement of the clinic's stance on antibiotic stewardship would also help to set pet owners' expectations to avoid dissatisfied experiences due to unmet demands for unnecessary antibiotics for their pets. Educational materials on AMR, like posters and brochures [13], can be placed in clinic waiting areas to educate pet owners and facilitate shared decision-making (SDM) with veterinarians during consultation [14,22]. Besides, clinics can also attract veterinary professionals who share similar AMR-related values to join them, fostering a community of practice to drive stewardship efforts.

### 4.2 Touchpoint #2: Diagnosis and deciding on an appropriate antibiotic treatment plan

Positive clinic dynamics, such as open communication amongst colleagues to share knowledge and best practices in antibiotic prescribing, were notable [23]. Unlike human primary care settings [13], veterinarians often sought inputs from veterinary nurses and technicians who, though not licensed veterinarians in Singapore, were similarly trained but not from universities recognised by Singapore's licensing conditions. This underscores the importance of offering collective training to all clinic staff, not just licensed veterinarians, to ensure alignment in knowledge and practices [13,23].

At the national level, the lack of contextualised training and local research evidence for appropriate antibiotic prescribing was identified as problematic, highlighting a need to establish a veterinary school in Singapore to provide tailored training relevant to local animal species. Encouraging conversations across different One Health sectors is crucial as well, aligning with Singapore's National Strategic Action Plan on AMR [24]. Regular continuing education talks amongst One Health professionals—covering animal, human, and environmental health—would be beneficial. Extending invitations to infectious disease conferences in human health to the veterinary community can foster the exchange of ideas and knowledge on AMR too [24].

### 4.3 Touchpoint #3: Shared decision-making and antibiotic prescribing

Similar to human medicine [25], unnecessary demand for antibiotics by pet owners was found to often stem from poor knowledge, and SDM can help address this issue and improve the appropriateness of prescribed antibiotics [13,26]. However, the unregulated sale of antibiotics by groomers, pet shops, and online sellers poses a challenge for veterinarians. Misuse of antibiotics in such settings is undocumented, and many pet owners may not be unaware that these actions can constitute misuse.

Veterinarians have called for tighter regulatory control to curb unauthorised antibiotic use. The establishment of a local veterinary council could effectively support this effort. Nonetheless, ongoing communication with pet owners should continue, extending further to educate and empower them to recognise and renounce unauthorised antibiotic use for their pets, aiding better antibiotic decision-making.

### 4.4 Touchpoint #4: Audit and feedback processes

Audit and feedback, alongside benchmarking, can enhance professional practices, including antibiotic prescribing behaviours [27,28]. For that to be effective, feedback should be provided through a supervisor or a co-worker for more than once, with targeted goals and actions recommended [27]. However, tracked indicators should be meaningful to the veterinarians, and SMART – specific, measurable, achievable, relevant and time-bound [29]. In veterinary settings, discussions between pet owners and veterinarians often revolve around considering ease of administration for pets to ensure compliance with recommended antibiotic doses [23]. Discussions surrounding costs were relatively uncommon, as supported by an article published by Coe *et al* [30]. Recognising SDM and tailoring antibiotic courses to pet owners' capacity to administer medication are important behaviours for veterinarians. Future audits should include these aspects. Technological advancements like big data and deep learning could offer potential solutions to concerns about resource limitations for data extraction and monitoring [31].

### 4.5 Strengths and limitations

This study marks the first in-depth local exploration of its kind and represents the initial application of the VALUE model outside its primary care context. Through qualitative analysis, we observed that the VALUE model can be adapted to the veterinary setting.

However, this study is not without limitations. Even though the interviewers introduced themselves as not part of the veterinary profession and that the interviews were conducted in a private setting, social desirability bias was inevitable. It might have influenced responses, but veterinarians were overall forthcoming about the challenges to addressing AMR in their clinics; though their criticisms were sometimes tempered, these were still expressed. Lastly, while purposive sampling with maximum variation was employed, it might not have ensured full representativeness amongst the veterinarians in Singapore. Nonetheless, the principle of data saturation was applied to confirm the themes that emerged and to draw the conclusions that were presented here.

## 5 Conclusion

Overall, our findings underscore the necessity to design future policy using tailored strategies at various touchpoints to enhance appropriate value-driven antibiotic prescribing for pet cats and dogs. There is a need for increased awareness and emphasis on AMR within the veterinary community at both national and clinic levels in Singapore. Establishing communities of practice that promote agreed-upon antibiotic knowledge and practices amongst various veterinary roles and professions, as well as amongst One Health professionals nationally, is crucial. Audit indicators should be relevant and meaningful for veterinarians in clinic settings, enabling effective monitoring and evaluation of antibiotic prescribing behaviours.

## Ethics approval and consent to participate

Ethical approval for this study was obtained (reference number: 2021/00769) and all methods in this study were performed in accordance with the relevant guidelines and regulations from the National Healthcare Group Domain Specific Review Board, Singapore. All participants had provided informed consent to participate in the study.

## Consent for publication

Not applicable.

## Funding

This work was supported by the National Centre for Infectious Diseases, One Health Antimicrobial Resistance Research Programme (OHARP-001).

## CRediT authorship contribution statement

**Huiling Guo:** Writing – original draft, Project administration, Methodology, Formal analysis. **Zoe Jane-Lara Hildon:** Writing – review & editing, Methodology, Formal analysis. **Lok Hang Wong:** Writing – review & editing, Formal analysis. **Timothy Chua:** Writing – review & editing. **Boon Han Teo:** Writing – review & editing. **Angela Chow:** Writing – review & editing, Supervision, Methodology, Funding acquisition, Conceptualization.

## Declaration of competing interest

All the authors declare no competing interests.

## Data Availability

The datasets used and/or analysed during the current study are available from the corresponding author on reasonable request.

## Appendix A Supplementary data

Supplementary material: Interview guide for veterinarians Image 1
