# Supplementary material for: The VALUE of antibiotic stewardship for companion animals: Understanding appropriate antibiotic prescribing for pet cats and dogs in veterinary clinics in Singapore
Source: One Health. 2025 Feb 11;20:100994. doi: 10.1016/j.onehlt.2025.100994 (PMC11875800; doi:10.1016/j.onehlt.2025.100994)
Supplement: Supplementary file 1 — Supplementary material: Interview guide for veterinarians [file mmc1.docx]

**INTERVIEW GUIDE FOR VETERINARIANS**

**Part A: To explore the context underlying antibiotic prescribing in the veterinary setting**

- What are the top 3 conditions which you will prescribe antibiotics for companion animals such as dogs and cats?
- What are the top 3 antibiotics prescribed for the conditions you have mentioned?
- How do you decide on whether antibiotics should be prescribed?
- How do you decide on the choice of antibiotics, dose and duration?

National level

- How is antibiotic prescribing guided in the veterinary setting?
  - Are there any guidelines which you would refer to when you need to prescribe antibiotics for companion animals such as cats and dogs?
- How do you feel about using guidelines, in general, to guide antibiotic prescribing?

Organisational level

- Would you mind sharing with us on what’s the organisational structure like in the veterinary clinic you are practicing in now?
- How many other professionals you have in your clinic (for example, other veterinarians, veterinary technicians and veterinary nurses)?
  - Have they ever influenced your antibiotic prescribing decisions?
- Would you mind sharing how pet owners pay for each consultation or service?
  - Do you think that pet owners have different expectations for the antibiotics when they pay out-of-pocket or insurance?
    - Why do you think so?
- In Singapore, there are more than 300 veterinarians registered and practicing in over 90 veterinary clinics. In such an environment, would you think that it is necessary to keep the business competitive in order to maintain a sustainable pool of clients?
  - Why would you think so?
  - What are some strategies used by your clinic?
- Would you mind sharing with us how drugs are procured and managed within your clinic?
- Understand that in some situations, pet owners may purchase drugs, including antibiotics, from pet shops, groomers or the internet for use on companion animals. Have you heard of such instances?
  - How do you feel about it?
- What’s the usual protocol for treating a suspected bacterial infection?
- Do you think that it is necessary for every antibiotic prescribed to be guided by diagnostic tests such as radiology or cultures?
  - Why do you think so?

**Part B: To explore the mechanisms influencing antibiotic prescribing in the veterinary setting**

Relationship building and understanding client’s perspectives

- Would you mind sharing how’s the daily clientele load in your clinic?
  - Do you often see back the same clients, i.e. there is a continuity of care by the same veterinarian, in your clinic?
  - Has seeing back or not seeing back the same clients influenced your antibiotic prescribing decision?
  - How about seeing the same companion animal but managing different owners at each consultation?
- Do you think this clientele load has influenced effective communication with the pet owners on antibiotic prescribing?
- Do you think this clientele load has influenced trust and relationship building with the pet owners on antibiotic prescribing?
- Would you mind sharing with us on your opinion of the knowledge, attitude and perception of pet owners on appropriate antibiotic use?

Alignment of organisational culture and related values with personal values toward appropriate antibiotic prescribing

- Do you own the practice you are practicing in now?
- Are you able to practice whatever you have envisioned yourself to be as a veterinarian in this current clinic?
- **[If participant owns practice]:** What kind of your personal values do you translate to your fellow employees?
- **[If participant is a locum]:** Would you mind elaborating on how organisation differences could influence the way you practice in different clinics?

Emphasis on liaison with clients for shared decision-making on antibiotic prescribing

- In this clinic you are practising in, is there an emphasis on shared decision-making with pet owners on antibiotic prescribing?
  - **[If yes]:** Is this part of your organisational value to do so? Or is it part of your personal value as a veterinarian?
  - **[If no]:** Why not?
    - Would you think that it is necessary for shared decision-making to take place with pet owners on antibiotic prescribing?
      - Why or why not?
  - Do you feel that pet owners would prefer shared decisions for antibiotics?
    - Why would you say so?

Barriers and facilitators of using data to monitor and evaluate appropriate antibiotic prescribing behaviors and related outcomes

- What are your thoughts of having a data monitoring or audit system in place on antibiotics prescribed in the veterinary clinics?
  - What do you think the role of antibiotic prescribing audits can be in veterinary practice?
